# HNF1B inhibits cell proliferation via repression of SMAD6 expression in prostate cancer

**DOI:** 10.1111/jcmm.16081

**Published:** 2020-11-10

**Authors:** Wei Lu, Jian Sun, Huihui Zhou, Fei Wang, Chunchun Zhao, Kai Li, Caibin Fan, Guanxiong Ding, Jianqing Wang

**Affiliations:** ^1^ School of Nursing Suzhou Vocational Health College Suzhou China; ^2^ Department of Urology The Affiliated Suzhou Hospital of Nanjing Medical University Suzhou China; ^3^ Department of Pathology Affiliated Yuhuangding Hospital of Qingdao University Yantai China; ^4^ Department of Urology Huashan Hospital Fudan University Shanghai China

**Keywords:** CDKN2A, Cyclin D1, HNF1B, proliferation, prostate cancer, SMAD6

## Abstract

Prostate cancer is the most common malignancy in men in developed countries. In previous study, we identified HNF1B (Hepatocyte Nuclear Factor 1β) as a downstream effector of Enhancer of zeste homolog 2 (EZH2). HNF1B suppresses EZH2‐mediated migration of two prostate cancer cell lines via represses the EMT process by inhibiting SLUG expression. Besides, HNF1B expression inhibits cell proliferation through unknown mechanisms. Here, we demonstrated that HNF1B inhibited the proliferation rate of prostate cancer cells. Overexpression of HNF1B in prostate cancer cells led to the arrest of G1 cell cycle and decreased Cyclin D1 expression. In addition, we re‐explored data from ChIP‐sequencing (ChIP‐seq) and RNA‐sequencing (RNA‐seq), and demonstrated that HNF1B repressed Cyclin D1 via direct suppression of SMAD6 expression. We also identified CDKN2A as a HNF1B‐interacting protein that would contribute to HNF1B‐mediated repression of SMAD6 expression. In summary, we provide the novel mechanisms and evidence in support HNF1B as a tumour suppressor gene for prostate cancer.

## 1 INTRODUCTION

In western countries, prostate cancer remains the leading cause of cancer‐related mortality among men.^1^ The development of prostate cancer mostly depends on the increased cell proliferation compared with healthy prostate tissue. There are several potential mechanisms that participate in prostate cancer progression through an influence on cell proliferation. Although many studies have focused on oncogenes, signal transduction pathways and cellular processes that promote cell proliferation in the progression of prostate cancer disease, the mechanism of prostate cancer cell proliferation is still not fully understood. Therefore, to further explore the mechanisms in prostate cancer cell proliferation is essential for deeply understanding of the development of other new therapeutic strategies.

Hepatocyte nuclear factor 1β (HNF1B) encodes a transcription factor that regulates important gene and metabolic pathway in vertebrate development and embryonic survival.^2^ In recent years, the results of genome‐wide association studies (GWAS) and fine‐mapping analysis have found that several different variants in the HNF1B gene are associated with the genetic risk of prostate cancer,^3^, ^4^, ^5^, ^6^, ^7^, ^8^, ^9^ which suggest that HNF1B has potential roles in prostate cancer progression. Our previous studies have shown that HNF1B, cooperating with RBBP7, serves as a transcriptional target of EZH2 and mediator of EZH2 prometastatic effects for the first time.^10^ HNF1B can inhibit the EMT process of prostate cancer cell via suppression of SLUG expression, resulting in the inhibition of distant metastasis. We also discover the critical role of HNF1B in prostate cancer cell growth,^11^ but the molecular mechanism by which is still not clear.

The Cyclin D1 protein encoded by the CCND1 gene can form a complex with CDK4 or CDK6, and can regulate related subunits. The activity of this complex is necessary for the cell cycle G1/S transition.^12^ Cyclin D1 promotes cell proliferation and correlates with early cancer onset and tumour progression in many cancer types, including prostate cancer. SMAD6 is an inhibitory Smad of the transforming growth factor‐beta 1 (TGF‐β1) superfamily. SMAD6 inhibits TGF‐β family signalling through its MH2 domain binding to type I receptors, thereby preventing the recruitment and phosphorylation of the effector SMADs1‐3.^13^ Previous studies have shown that TGF‐β1 inhibits Cyclin D1 in prostate cancer and other cancer types, in order to constrain prostate cancer growth and metastatic progression.^14^, ^15^

In this study, we found that the overexpression of HNF1B in prostate cancer cells can inhibit cell growth and lead to G1 cell cycle arrest through in vivo and in vitro experiments. In mechanism research, we determined that HNF1B inhibits prostate cancer cell proliferation by down‐regulating the expression of Cyclin D1. Further investigation indicated that HNF1B, interacting with CDKN2A, suppressed Cyclin D1 expression via direct inhibition of SMAD6. Taken together, our data suggest that the role and mechanisms of HNF1B in prostate cancer proliferation make it a potential therapeutic target of this highly aggressive tumour.

## 2 MATERIALS AND METHODS

### 2.1 Cell lines and cell culture

PC‐3 and DU145 cells were maintained in RPMI1640 supplemented with 10% FBS. All cells were supplemented with an antibiotic solution (100 units/mL penicillin and 0.1 mg/mL streptomycin) and grown at 37°C in standard cell culture conditions (5% CO2, 95% humidity). Control, HNF1B and CCND1 stable cells were obtained by co‐transfection of DU145 or PC‐3 cells with pPB‐CAG‐ires‐Pac, pPB‐CAG‐HNF1B‐ires‐Pac or pPB‐CAG‐CCND1‐ires‐Pac and pCMVPBase. After 2 μg/mL puromycin (Amresco) screening for 2 weeks, stable cell lines were selected and identified by Western blotting. SMAD6 overexpression (OE) HNF1B‐DU145 cells were obtained by transfection of HNF1B‐DU145 cells with pPB‐CAG‐SMAD6‐ires‐Pac.

### 2.2 Constructs

pPB‐CAG‐ires‐Pac was generated as previously described.^16^ pPB‐CAG‐HNF1B/ SMAD6/ CCND1‐ires‐Pac was generated by ligating full length HNF1B, CCND1 and SMAD6 into the multiple‐cloning sites (MCS) of pPB‐CAG‐ires‐Pac.

### 2.3 siRNA transfections

In this study, we tested the individual set of 3 siRNAs (GenePharma) against HNF1B by Western blotting. The effective single siRNAs (GenePharma) against HNF1B were used for further experiments. For siRNA transfection in 6‐well plates, 3 × 10^5^ cells per well were subjected to reverse transfection with 100 nM siRNA (GenePharma) using Lipofectamine 2000 transfection reagent (Invitrogen), following the manufacturer's instructions.

### 2.4 Antibodies and immunoblotting

Cells were lysed using 1× SDS loading buffer (50 mM Tris‐HCl pH6.8, 10% glycerol, 2% SDS, 0.05% bromophenol blue and 1%2‐mercaptoethanol).Antibodies were listed as follows: anti‐HNF1B antibody (Proteintech), anti‐Cyclin D1 (610 181, BD Transduction Laboratories), anti‐SMAD6, anti‐p‐SMAD2/3, anti‐FLAG (Proteintech), anti‐Vinculin (Proteintech) and anti‐tubulin (Abcam). For immunoblot, proteins were separated by SDS–PAGE and transferred to polyvinylidene difluoride membranes (Millipore). HRP‐conjugated secondary antibodies (Jackson laboratories) and enhanced chemiluminescence system were used for signal detection. Protein was visualized using KODAK film machine or ChemiDoc XRS chemiluminescence detection and imaging system (Bio‐Rad Laboratories).

### 2.5 Immunohistochemistry

Immunohistochemistry (IHC) was performed as previously described.^17^ In short, the streptavidin‐biotin‐peroxidase method was used to perform immunohistochemistry with diaminobenzidine as the chromogen. Two blinded, independent observers (including a pathologist) simultaneously examined HNF1B and Cyclin D1 staining, and each core reached a consensus score. The staining pattern is defined as: 0, negative; 1‐2, weak; 3‐4, medium; 5‐6, strong.

### 2.6 Cell proliferation assay

The MTS assay was performed as previously described.^10^ Briefly, all cells were implanted in 96‐well plates at a density of 4000 cells/well and incubated at 37°C for a total of 6 days. Then 100 μL of RPMI‐1640 medium containing 20 μL of CellTiter 96 AQueous One solution was added to each well and incubated for 1 hour. The cell number was estimated by monitoring the absorbance at 490 nm using a SYNERGY HT microtiter plate reader (Bio‐tek).

### 2.7 In Vivo Tumour Growth Assay

Six‐week‐old male athymic mice were inoculated s. c. with 100 μL of a mixture containing 1 × 10^7^ prostate cancer cells. Tumour growth was monitored weekly, and mice were sacrificed after 4 weeks. Tumours were weighted and tumour sizes were measured recorded in mm^3^ (length × width^2^). Our research was approved by the Ethics Committee of Nanjing Medical University and followed all the principles of human or animal experimental research outlined in the Declaration of Helsinki.

### 2.8 Colony formation assay

The colony formation assay was performed as previously described.^16^ Briefly, control and HNF1B stably overexpressed DU145 and PC‐3 cells were planted in six‐well plates at 1000 cells/well (in triplicate) and cultured at 37°C and 5% for 7‐10 days. Colonies were fixed with 4% (w/v) paraformaldehyde for half an hour and stained with crystal violet (Sigma‐Aldrich) for 20 minutes. After that, EPSON Imager 600RGB was used to take photographs of the colonies. Colony quantification was performed using Ipwin32 software.

### 2.9 Real‐time RT‐PCR

Total RNA was extracted from cells using TRIzol reagent (Invitrogen). We then followed the manufacturer's instructions (Fermentas) to reverse transcription of RNA using reverse transcriptase. The Bio‐Rad CFX96 system was used for quantitative real‐time PCR, and the relative gene expression was normalized to an internal control (gapdh). Primer sequences of target genes were shown as follows:

SMAD6 (forward) 5′‐CAAGCCACTGGATCTGTCCGA‐3′;

(reverse) 5′‐TTGCTGAGCAGGATGCCGAAG;

CCND1 (forward) 5′‐CGTGGCCTCTAAGATGAAGG‐3′,

(reverse)5′‐CTGGCATTTTGGAGAGGAAG‐3′;

### 2.10 Fluorescence‐activated cell sorting analysis

After trypsinization and resuspension in ice‐cold buffer (30% phosphate buffer, 70% ethanol) at a concentration of 1‐5 × 10^6^ cells/mL, the cells can grow to 70%‐80% confluence for at least 1 hour. The fixed cells were recovered by centrifugation (1000 *g*, 5 minutes), washed 3 times in PBS, and resuspended in 1 mL PBS containing 8 mg RNaseA and 50 mg propidium iodide (PI). The PI‐stained samples were then incubated for 30 minutes in the dark at room temperature and measured by FACS on the BD FACS Calibur using Cellquest software.

### 2.11 Publicly available gene expression data sets and clinical data sets

In this article, we use some publicly available individual prostate cancer clinical data sets. These include the data reported in GSE21032 and a similar set of prostate adenocarcinoma samples from cBioPortal Cancer Genomics (The Cancer Genome Atlas, TCGA‐provisional).^18^

### 2.12 IP‐mass spectrometry

Assays were performed as previously described.^10^ For IP, the cell lysate was incubated with the marker antibody for 3 hours at 4°C, then protein A‐Sepharose beads (GE Healthcare) were added to the mixture, and then incubated at 4°C for 1 hour. The IP complex was washed five times with NETN buffer. After washing, the IP samples were separated by SDS‐PAGE on a 4%‐20% polyacrylamide gel (Bio‐Rad) and visualized using Bio‐Safe Coomassie staining (Bio‐Rad). The gel containing the FLAG‐HNF1B complex was cut out and treated with dithiothreitol to reduce disulphide bonds and iodoacetamide to alkylate cysteine. In‐gel digestion of proteins treated with trypsin. The peptides are extracted from the gel and analysed by liquid chromatography tandem mass spectrometry. Based on quality accuracy, trypsin status (for trypsin) and XCorr will filter all peptides and confirm by manual inspection.

### 2.13 Statistical analysis

The results are represented as the average values ± standard error of mean (SEM). For RNA‐seq data re‐exploration, we used EdgeR to identify differential expressed genes (DEGs) as the following criterion: fold change (FC) ≥2 or ≤ 0.5; *P* value <.01. Between‐group variations were evaluated by the use of the Student's *t* test. Spearman's correlation finished the exploration of the relationships. A *P* value below .05 was thought to have statistical significance.

## 3 RESULTS

### 3.1 Forced expression of HNF1B suppresses cell growth of prostate cancer cell lines and resulted in G1‐phase cell cycle arrest

**Figure 1:**
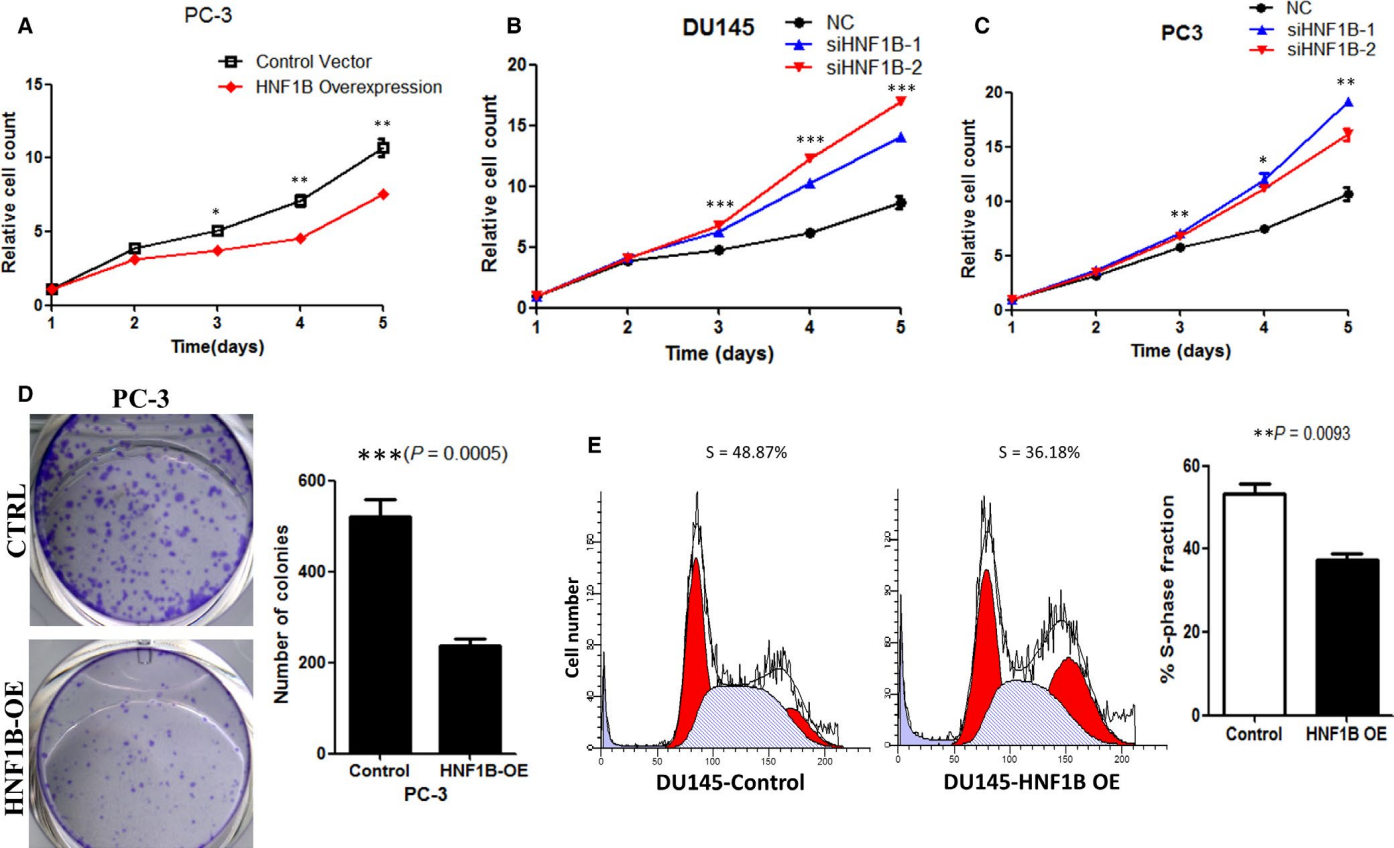
HNF1B suppresses prostate cancer cell growth via G1‐phase cell cycle arrest. (*P < .05, **P < .01, ***P < .001). A, Forced expression of HNF1B suppresses cell growth of PC‐3. B, Cell proliferation of HNF1B knockdown in DU145 was measured at the indicated time points. C, Cell proliferation of HNF1B knockdown in PC‐3 was measured at the indicated time points. D, Results of colony formation assay in PC‐3 cells transfected with control vector and HNF1B. E, The effect of HNF1B on cell cycle

In our previous study, we have shown that HNF1B can diminish the effect of EZH2 in promoting prostate cancer proliferation. We also showed the role of HNF1B alone on proliferation in DU145 cells.^11^ To further confirm the effect of HNF1B in prostate cancer cell proliferation, we overexpressed HNF1B in PC‐3 cells and knocked down HNF1B in both PC‐3 and DU145 cells. Stable cell lines were used as in previous manuscript (data not shown). Cell proliferation assays showed that forced expression of HNF1B significantly inhibited PC‐3 cell growth. Conversely, loss of HNF1B significantly promoted cell growth in PC‐3 and DU145 cells. All the results were consistent with our previous results in DU145 (Figure 1A‐C).

Then, we detected the colony formation ability after HNF1B overexpressing in prostate cancer cell lines. After 7‐day observation, we found that the clonogenic ability of prostate cancer cells was suppressed in HNF1B overexpressed PC‐3 cells, which was also consistent with our previous results in DU145 (Figure 1D).

To study the mechanisms by which HNF1B inhibits proliferation of prostate cell lines, we first analysed cell cycle profile in HNF1B overexpressed DU145 cells. HNF1B overexpression resulted in a decreased proportion of cells in S phase (Figure 1E), which indicated that HNF1B suppressed prostate cancer cell proliferation by influencing cell cycle through G1‐phase cell cycle arrest.

### 3.2 HNF1B regulates Cyclin D1 expression in prostate cancer cells

**Figure 2:**
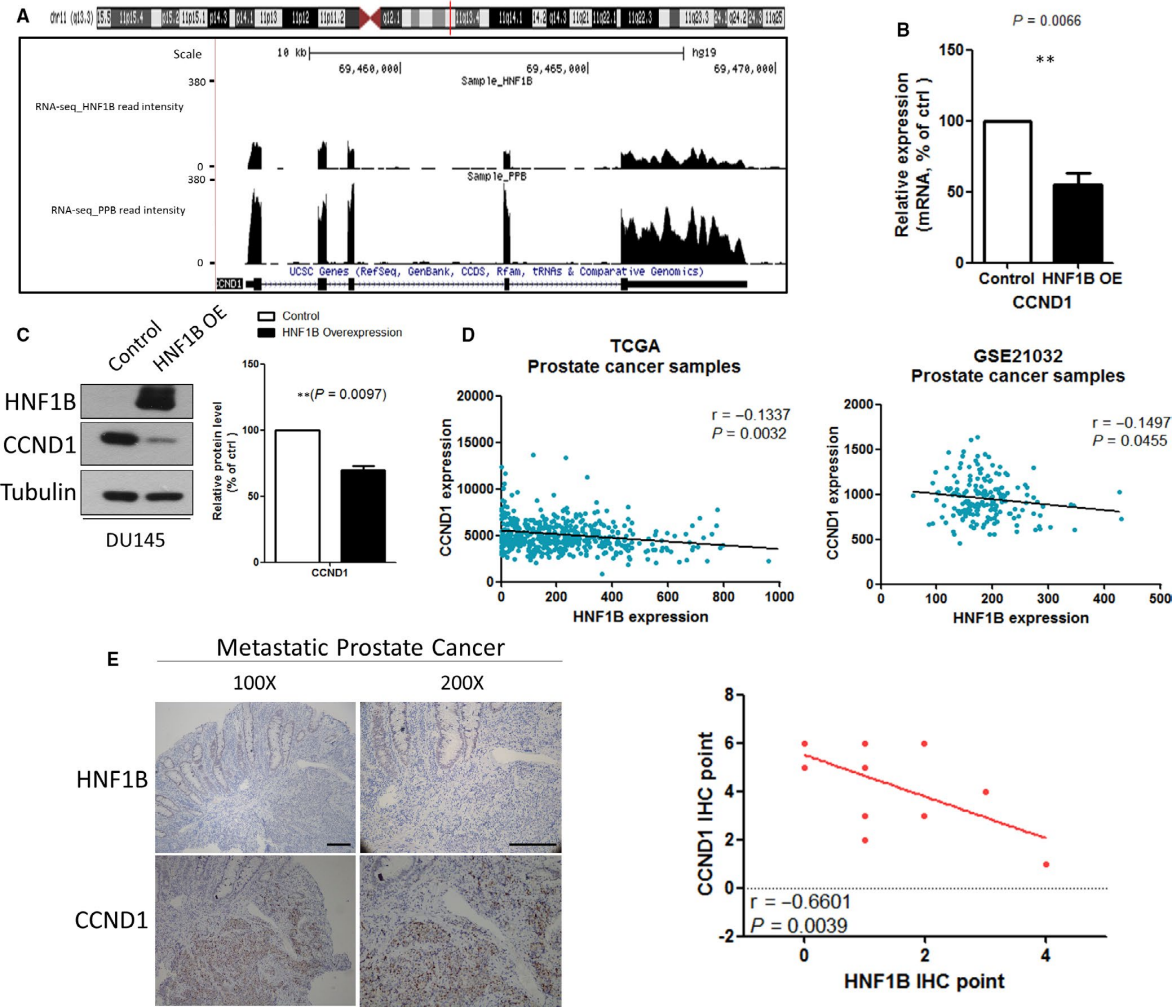
HNF1B regulates cyclin D1 expression in prostate cancer cells. A, A representative HNF1B RNA‐seq peaks located on CCND1. B, mRNA levels of CCND1 in control and HNF1B‐OE DU145 cells. C, Representative Western blotting analysis of HNF1B inhibiting CCND1 in DU145 cells. D, Expression correlation analyses of CCND1 and HNF1B mRNA levels in prostate cancer samples from TCGA and GSE21032. E, Representative IHC staining of CCND1 and HNF1B protein levels in metastatic prostate cancer tissue

Then we re‐explored data of RNA‐seq from HNF1B overexpressed and control DU145 cells in our previous study. We set the criterion of DEGs to fold change (FC) ≥2 or ≤ 0.5 and *P* < .01. Among the DEGs, we found that Cyclin D1 (CCND1) down‐regulated significantly after HNF1B overexpression (Figure 2A, Table S1). To confirm the result of RNA‐seq, we then performed RT‐PCR and Western blotting analysis and confirmed that increased expression of HNF1B led to decreased Cyclin D1 expression in both mRNA and protein levels in both DU145 and PC‐3 cells (Figure 2B,C, Figure S1A). Cyclin D1 is a key regulator of cell cycle, which promotes cell proliferation and is amplified or overexpressed in several types of human cancer, including prostate cancer. This finding suggested the possibility that HNF1B inhibited prostate cancer cell proliferation through the suppression of Cyclin D1 expression.

To further confirm the correlation between HNF1B and Cyclin D1 expression in prostate cancer samples, we first analysed online publicly available data sets from TCGA data set and GSE21032. All data from the datasets above showed that the mRNA expression levels of HNF1B and Cyclin D1 were inversely correlated with a Pearson correlation coefficient of −0.1337 and −0.1497 (*P* = .0032 and *P* = .0455; Figure 2D). Then we did immunohistochemistry and found significant reverse correlation between HNF1B and Cyclin D1 protein levels in metastatic prostate cancer tissues (Figure 2E).

### 3.3 Forced expression of Cyclin D1 reverses HNF1B‐mediated cell growth inhibition

**Figure 3:**
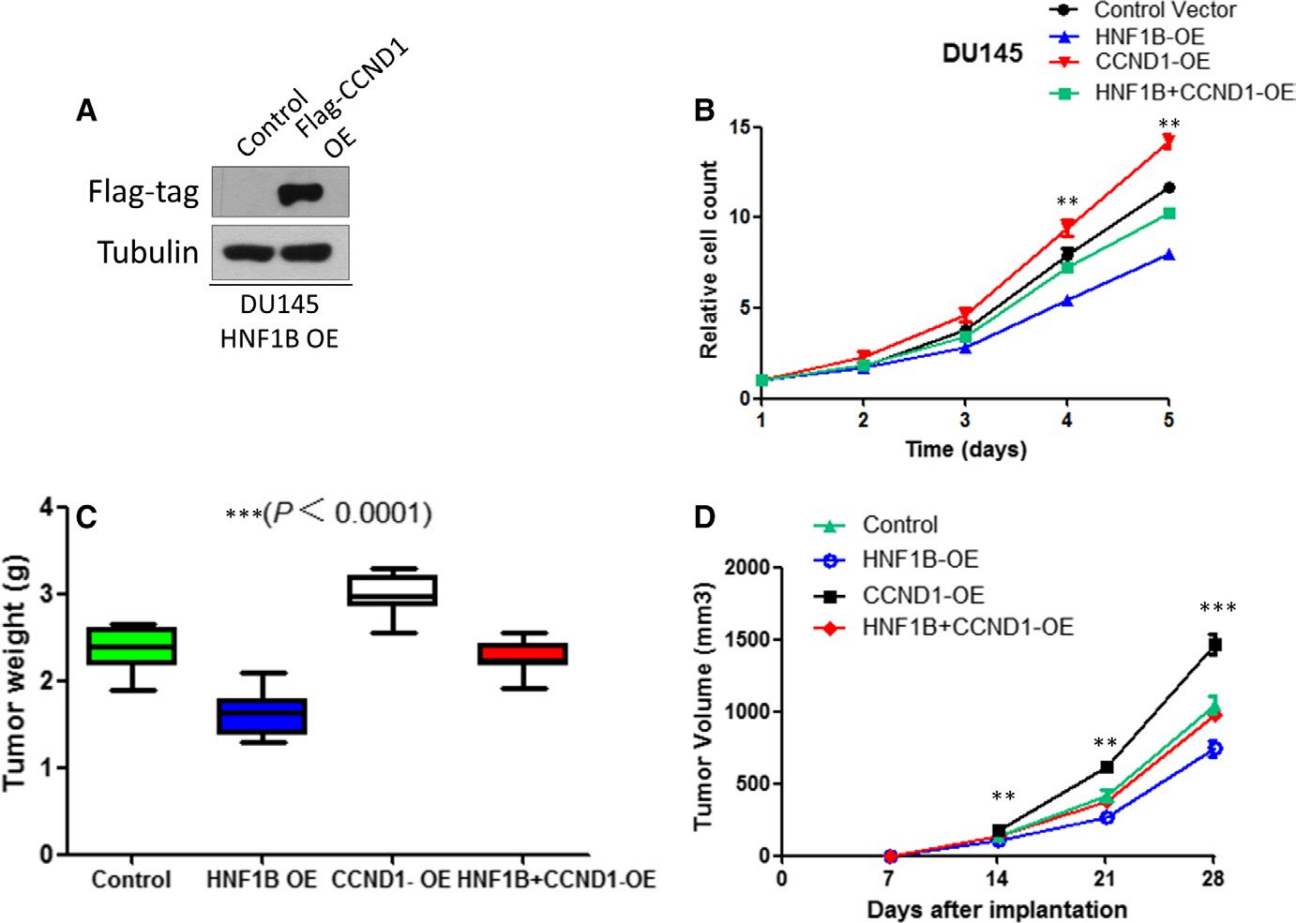
Forced expression of Cyclin D1 reverses HNF1B‐mediated cell growth inhibition. A, Western blot showed CCND1 overexpression in DU145. B, Cell proliferation was measured at the indicated time points in control, HNF1B‐OE, CCND1‐OE and HNF1B + CCND1‐OE DU145 cells (*P < .05, **P < .01, ***P < .001). C and D, Xenograft analyses of control, HNF1B‐OE, CCND1‐OE and HNF1B + CCND1‐OE DU145 cells (Scale bar, 1 cm, **P < .01, ***P < .001)

Cyclin D1 is a major cell cycle regulator for both normal and tumour cells. To determine if Cyclin D1 loss resulted in HNF1B‐mediated proliferation inhibition, we tested whether forced expression of Cyclin D1 could relieve the phenotype. We first overexpressed Cyclin D1 in HNF1B‐OE‐DU145 cells. Western blotting showed Cyclin D1 was up‐regulated significantly in Cyclin D1 overexpression cells (Figure 3A). Then, we measured cell growth for up to six days. As expected, overexpression of Cyclin D1 successfully mitigated HNF1B‐mediated cell growth inhibition of DU145 prostate cancer cells (Figure 3B). Results of in vivo xenograft tumour model of DU145 cells also revealed that up‐regulation of Cyclin D1 expression rescued the growth inhibition of tumour after HNF1B overexpression (Figure 3C,D).

All these data above indicate that HNF1B suppresses the proliferation of prostate cancer cells by inhibiting the expression of Cyclin D1.

### 3.4 HNF1B suppresses Cyclin D1 via direct repression of SMAD6

To figure out the mechanisms how HNF1B suppresses Cyclin D1 expression, we re‐explored the genes that HNF1B directly binds to and that also differentially expressed in ChIP‐seq and RNA‐seq. After analysis, we focused on SMAD6, which is a classic inhibitory Smad (I‐Smad) that can directly bind to activated TGF‐β Receptor‐I and block the receptor activation of R‐Smad. Previous studies have shown that SMAD6 can regulate or inhibit the gene expression by inhibiting TGF‐β pathway activity.^19^ In prostate cancer, TGF‐β inhibits proliferation by prevention of G1 progression in early stages of prostate cancer cells via suppressing Cyclin D1 mRNA and protein expression.^14^ Therefore, we wonder whether HNF1B inhibits Cyclin D1 through the suppression of SMAD6.

**Figure 4:**
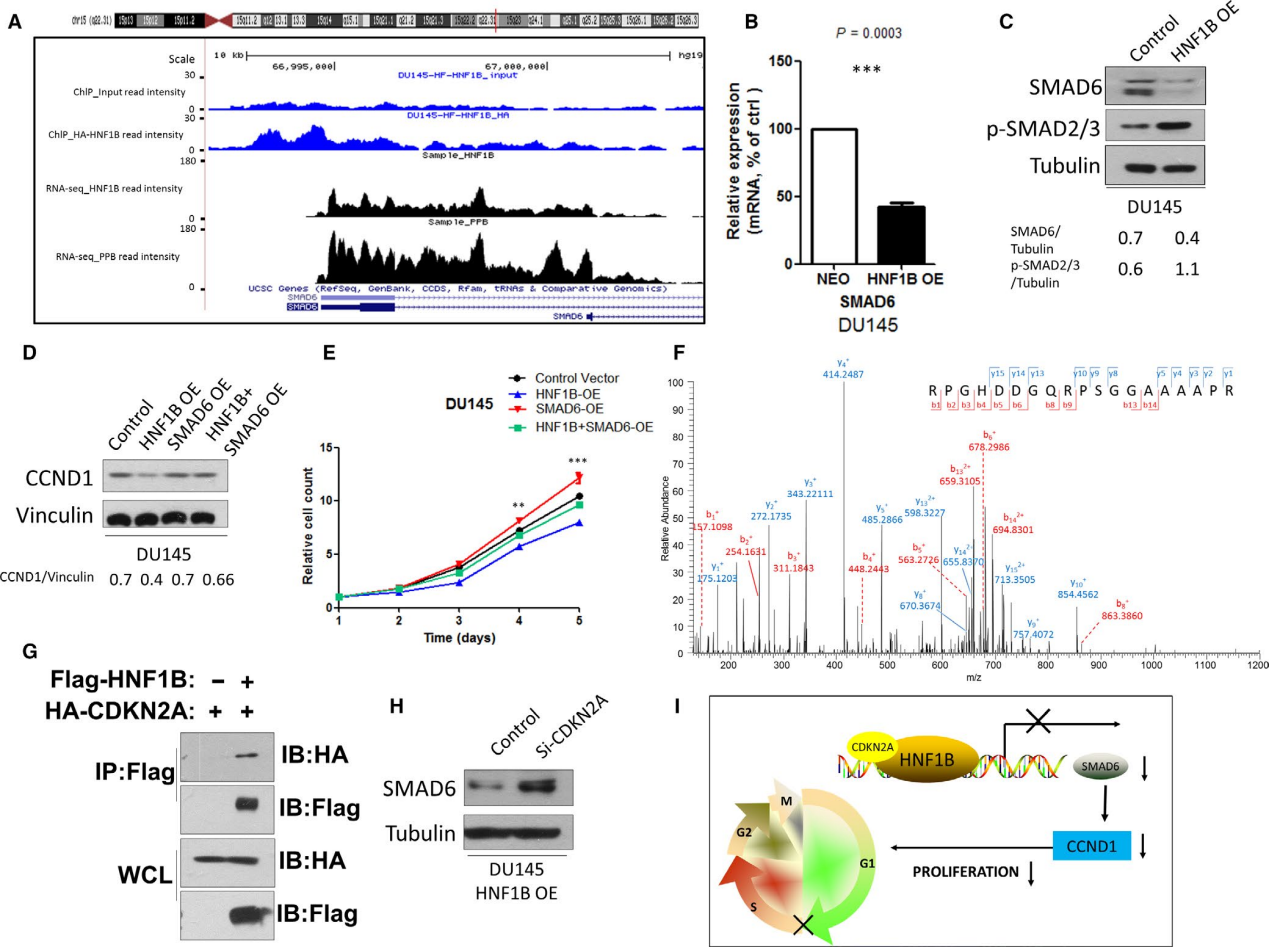
HNF1B suppresses Cyclin D1 via direct repression of SMAD6 with CDKN2A. A, A representative HNF1B ChIP‐seq peak and RNA‐seq peaks located on SMAD6. B, mRNA levels of SMAD6 in control and HNF1B‐OE DU145 cells. C, Western blot showed SMAD6 and p‐SMAD2/3 expression levels in HNF1B‐OE DU145 cells. D, Western blot showed CCND1 expression level in control, HNF1B‐OE, SMAD6‐OE and HNF1B + SMAD6‐OE DU145 cells. E, Cell proliferation was measured at the indicated time points in control, HNF1B‐OE, SMAD6‐OE and HNF1B + SMAD6‐OE DU145 cells (*P < .05, **P < .01, ***P < .001). F, Mass spectrometry identification of CDKN2A peptide RPGHDDGQRPSGGAAAAPR. G, Co‐IP analysis of the interaction between HNF1B and CDKN2A. H, CDKN2A knockdown induced SMAD6 up‐regulation in HNF1B‐OE DU145 cells. I, Working model depicting the mechanism of HNF1B/CDKN2A‐mediated SMAD6 suppression in prostate cancer

Results of ChIP‐seq and RNA‐seq indicated that SMAD6 was down‐regulated in HNF1B overexpressed DU145 cells and that HNF1B bound to its promoter‐TSS region (Figure 4A). RT‐PCR and Western blotting analysis indicated the decreased SMAD6 expression in HNF1B overexpressed DU145 and PC‐3 cells (Figure 4B,C, Figure S1B). Furthermore, to determine if TGF‐β signalling activity was influenced after HNF1B overexpression, we tested the induction of SMAD2/3 phosphorylation as this could be regulated by the TGF‐β pathway.^20^, ^21^ In HNF1B OE DU145 cells, SMAD2/3 was hypophosphorylated compared with the control cells (Figure 4C).

Moreover, we re‐introduced SMAD6 to see whether this can rescue Cyclin D1 expression and the inhibition of proliferation. As expected, overexpression of SMAD6 significantly increased Cyclin D1 expression in HNF1B overexpressed DU145 and PC‐3 cells (Figure 4D, Figure S1C,D). Forced expression of SMAD6 completely mitigated HNF1B‐mediated cell growth inhibition of DU145 prostate cancer cells (Figure 4E). All these data together demonstrated that SMAD6 is a direct target of HNF1B in the regulation of Cyclin D1 expression and cell growth of prostate cancer cells.

### 3.5 HNF1B suppresses SMAD6 expression by interacting with CDKN2A

In the last step, we tried to discover the mechanisms how HNF1B could mediate transcription repression. We then explored the potential HNF1B interacting proteins in mass spectrometry analyses before, and focused on CDKN2A (P16, Cyclin‐Dependent Kinase Inhibitor 2A, Figure 4F). CDKN2A is a tumour suppressor that inhibits cell proliferation via various mechanisms. Using co‐immunoprecipitation (co‐IP) analysis, the interaction between Flag‐HNF1B and HA‐CDKN2A was confirmed in HEK293T cells (Figure 4G). In addition, we also found that CDKN2A was required for HNF1B‐mediated suppression of SMAD6 expression, because the knockout of CDKN2A resulted in increased SMAD6 expression even if HNF1B was overexpressed (Figure 4H).

## 4 DISCUSSION

HNF1B encodes a transcription factor and acts as a central regulator in vertebrate development and embryonic survival.^2^ Recent years, genome‐wide association studies (GWAS) and fine‐mapping analysis have identified several distinct variants within HNF1B gene associated with increased risk of prostate cancer, such as rs11649743 and rs4430796,^3^, ^4^, ^5^, ^6^, ^7^, ^8^, ^9^ which suggest that HNF1B has potential roles in prostate cancer progression. Our previous studies showed the preliminary function of HNF1B in prostate cancer proliferation and migration as a potential tumour suppressor gene. However, the precise roles and mechanisms of HNF1B in prostate cancer proliferation are still not clear. In previous study, we have identified HNF1B as a transcriptional target of EZH2 that can rescue EZH2‐mediated migration and cell growth in prostate cancer. We also demonstrate that HNF1B directly suppresses SLUG gene transcription and expression to repress EMT progress and cell migration.^10^ However, the molecular mechanisms involved in how HNF1B influences the proliferation of prostate cancer cells are still not well understood.

In the present study, we demonstrate that HNF1B inhibits prostate cancer cell proliferation by suppressing Cyclin D1 expression. HNF1B could interact with CDKN2A, and directly bind and down‐regulate SMAD6 expression level, which leads to the activation of TGF‐β signalling. In prostate cancer, TGF‐β inhibits proliferation by prevention of G1 progression in early stages of prostate cancer cells via suppressing Cyclin D1 mRNA and protein expression (Figure 4I).

To dig out how HNF1B represses prostate cancer cell proliferation, we analysed cell cycle of HNF1B OE DU145 cell. Results indicate that forced expression of HNF1B results in G1‐phase cell cycle arrest. To further elucidate the mechanism how HNF1B influences the cell cycle, we re‐explored and analysed the RNA‐seq data, and focused on Cyclin D1, which a key regulator of cell cycle (G1‐ phase) for both normal and tumour cells, is negatively regulated by HNF1B in prostate cancer cells. RT‐PCR and Western blotting both showed low expression of Cyclin D1 in HNF1B‐DU145 cells. Meanwhile, we interrogated publicly available microarray expression data sets online, results from which showed that the mRNA expression levels obtained for both HNF1B and Cyclin D1 were inversely correlated.

In addition, we alleviated HNF1B‐mediated cell growth inhibition of prostate cancer cells by overexpression of Cyclin D1 both in vitro and in vivo. Our data showed that the rescue of cell growth in HNF1B‐DU145 cells by Cyclin D1 is not due to decreasing HNF1B expression. Thus, Cyclin D1 is a functional target of HNF1B in prostate cancer cells. Then we wonder how HNF1B represses Cyclin D1 expression. To elucidate this issue, we combined the data of ChIP‐seq and RNA‐seq and found that SMAD6 is the direct downstream of HNF1B.

Smad6 is classified as inhibitory Smad (ISmad), because its interaction with the activated type I receptors prevents phosphorylation of the R‐Smads.^22^, ^23^, ^24^, ^25^, ^26^ SMAD6 is thought to play a role in the regulation of TGF‐β–mediated growth inhibition.^27^ TGF‐β1 has been shown to inhibit Cyclin D1 in order to constrain prostate cancer growth and metastatic progression.^14^ Then we put forward the hypothesis that HNF1B represses Cyclin D1 by suppression of SMAD6. To verify this issue, we investigated SMAD6 expression and TGF‐β signalling activity in HNF1B‐DU145 cells. As expected, HNF1B down‐regulated SMAD6 expression and thus induced TGF‐β signalling activity. Re‐introduction of SMAD6 restored Cyclin D1 expression and cell proliferation, which supports the hypothesis that HNF1B inhibits prostate cancer cell proliferation by direct repression of SMAD6.

In further research, we found that HNF1B can interact with CDKN2A, thereby inhibiting SMAD6 expression. CDKN2A is an important molecule in cells that regulates cells, and its loss has proved to be an important event in many types of cancer.^28^ CDKN2A can also induce cell cycle arrest in G1 phase.^29^ Previous studies have confirmed that it binds to MDM2 and prevents its nucleocytoplasmic shuttle by chelating it in the nucleolus, and inhibits the carcinogenic effect of MDM2 by preventing MDM2‐induced p53 degradation and enhancing p53‐dependent transactivation and apoptosis.^30^ CDKN2A could also inhibit cell growth promoted by MYC. CDK4 can inhibit the activity of CDKN2A, which is a transcription target of MYC.^31^ Moreover, other researchers showed the relationship between CDKN2A and other five genes and built up a CDKN2A‐centric network regulation which might exist in pancreatic ductal adenocarcinoma (PDCA).^32^ HNF1B mutation might affect the interaction with CDKN2A, and this might depend on the location of the mutation. Sense mutations located at the protein binding site may have a greater impact on the interaction with CDKN2A, while mutations located at other sites may have other effects. Our results provide new mechanisms how CDKN2A influences cell proliferation in prostate cancer.

Our results showed the roles of HNF1B‐CDKN2A in prostate cancer cell proliferation and related mechanisms. Our results revealed a new mechanism of HNF1B in prostate cancer development, and provided new understanding of the signalling regulation network of disease progression. Maybe in the near future, therapeutic drugs targeted HNF1B‐CDKN2A will come out, which could become potential drugs for the treatment of prostate cancer.

Our results have some limitations. We only demonstrated that HNF1B could bind CDKN2A to exert its role but did little on the interaction of HNF1B with CDKN2A. Indeed, there may be many mechanisms involved in the function of HNF1B to CDKN2A, which might involve more protein structure and function studies. Another point, mutations in HNF1B might affect the interaction with CDKN2A, this mostly depends on the location of the mutation. Sense mutations located at the protein binding site may have a greater impact on the interaction with CDKN2A. We will further investigate these issues above in the following study.

## 5 CONCLUSIONS

In conclusion, we have shown that SMAD6 is a direct target of HNF1B. HNF1B can down‐regulate SMAD6 expression, which results in the suppression of Cyclin D1 and cell proliferation inhibition. The observed interaction between HNF1B with CDKN2A contributes to suppression of SMAD6 and TGF‐β signalling, which suggests that HNF1B may play important roles in prostate cancer initiation and progression.

## CONFLICT OF INTEREST

The author(s) declared no potential conflicts of interest with respect to the research, authorship and/or publication of this article.

## AUTHOR CONTRIBUTIONS

**Wei Lu:** Data curation (equal); Methodology (equal); Writing‐original draft (equal). **Jian Sun:** Data curation (equal); Formal analysis (equal); Software (equal); Writing‐original draft (equal). **Huihui Zhou:** Investigation (equal); Methodology (equal); Resources (equal); Software (equal). **Fei Wang:** Data curation (equal); Formal analysis (equal); Resources (equal); Software (equal). **Chunchun Zhao:** Data curation (equal); Methodology (equal). **Kai Li:** Data curation (equal); Formal analysis (equal). **Caibin Fan:** Resources (equal); Software (equal). **G. Ding:** Data curation (equal); Project administration (equal); Resources (equal); Writing‐review & editing (equal). **Jianqing Wang:** Conceptualization (equal); Funding acquisition (lead); Investigation (equal); Project administration (equal).

## ETHICAL APPROVAL

The authors state that they have been approved by the Ethics Committee of Nanjing Medical University and have followed the principles outlined in the Declaration of Helsinki for all human or animal experimental investigations.

## Supporting information

Figure S1

Table S1

## Data Availability

The data that support the findings of this study are available in the [Link], [Link] of this article.
