# Supplementary figures and images for: HNF1B inhibits cell proliferation via repression of SMAD6 expression in prostate cancer
Source: J Cell Mol Med. 2020 Nov 10;24(24):14539–48. doi: 10.1111/jcmm.16081 (PMC7754016; doi:10.1111/jcmm.16081)

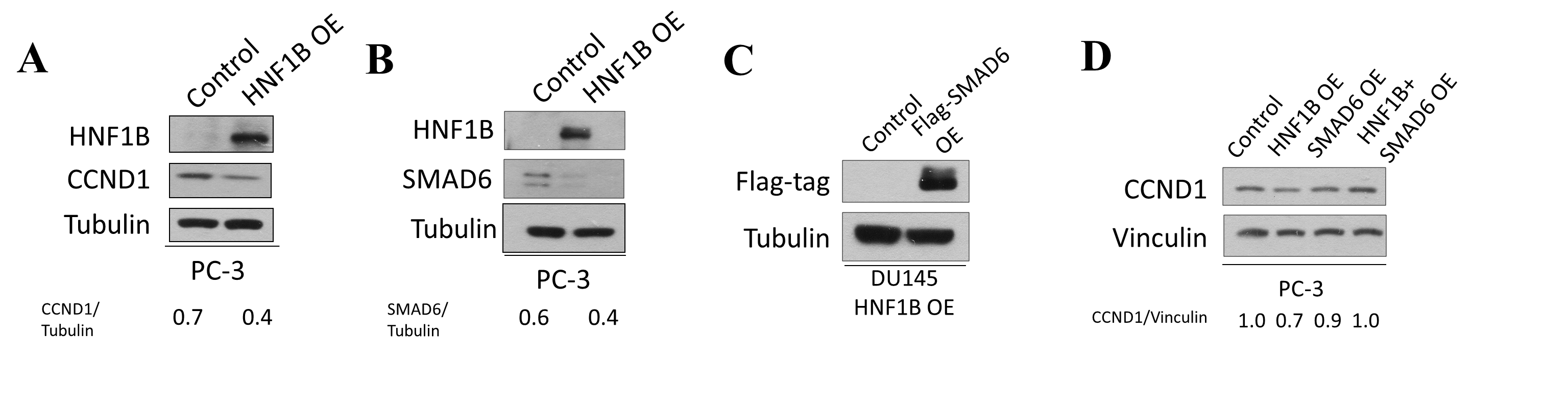

Supplement: Supplementary file 1 — Figure S1 [file JCMM-24-14539-s001.TIF]
